# Supplementary material for: WordCommentsAnalyzer: A windows software tool for qualitative research
Source: F1000Res. 2018 Sep 4;7:536. Originally published 2018 May 3. [Version 2] doi: 10.12688/f1000research.14819.2 (PMC6137411; doi:10.12688/f1000research.14819.2)
Supplement: Supplementary file 3 [file f1000research-7-17614-s0002.tgz › b2a79496-4ada-47c4-9682-a99ce867836d.zip!/Eight Iranian Health Journals/Journal of Education and Community Health_15.docx]

© Journal of Education and Community Health. This work is licensed under CC BY-NC 4.0 (https://creativecommons.org/licenses/by-nc/4.0/).

Volume & Issue: دوره 1 - شماره 2

Title: تأثیر مداخله آموزشی مبتنی برالگوی پرسید بر ارتقای کیفیت زندگی سالمندان

http://jech.umsha.ac.ir/article-1-39-fa.html

مقدمه: افزایش جمعیت سالمندان لزوم توجه به ارتقای کیفت زندگی آنها را افزایش داده است. مطالعه حاضر با هدف بررسی تأثیر مداخله آموزشی بر اساس الگوی پرسید در ارتقای کیفیت زندگی سالمندان انجام گرفته است.  مواد و روش ها: این مطالعه نیمه تجربی بر روی 80 نفر از سالمندان که به صورت تصادفی به دو گروه آزمون و کنترل تقسیم شده بودند، انجام شد. طراحی برنامه مداخله مطابق با مراحل الگوی پرسید صورت گرفت. قبل از اجرای مداخله پرسشنامه سنجش کیفیت زندگی و پرسشنامه خود ساخته و استاندار مطابق با مراحل الگوی پرسید در هر دو گروه تکمیل شد و یک ماه بعد از مداخله هر دو گروه مجدداً پرسشنامه های مذکور را تکمیل نمودند. داده های جمع آوری شده با استفاده از نرم افزار SPSS ویرایش 21 و آزمون های تی زوجی، تی مستقل، آنالیز واریانس یکطرفه، کای دو و آنالیز کوواریانس مورد تجزیه و تحلیل قرار گرفت.  یافته ها: میانگین نمره کل کیفیت زندگی در گروه آزمون قبل از مداخله آموزشی 13.23±67.77 بود که بعد از مداخله آموزشی به 12.25±82.87 افزایش یافت، بر این اساس اختلاف آماری معنی داری بین نمره کیفیت زندگی در دو گروه آزمون و کنترل بعد از مداخله آموزشی وجود داشت (0.031=P). یافته های حاصل از ابعاد مختلف کیفیت زندگی نیز نشان داد که اختلاف معناداری در ابعاد سلامت جسمی، روانی، محیطی و اجتماعی قبل و بعد از مداخله آموزشی وجود دارد (0.05>P).  نتیجه گیری: بکارگیری الگوی پرسید می تواند منجر به شناسایی عوامل تأثیر گذار بر کیفیت زندگی سالمندان و در نتیجه تدوین برنامه آموزشی مؤثر گردد.

Title: میزان تبعیت دختران دبیرستانی شهر همدان از برنامه پنج نوبت مصرف میوه و سبزیجات: کاربرد تئوری رفتار برنامه ریزی شده

http://jech.umsha.ac.ir/article-1-38-fa.html

مقدمه: یکی از ارکان تغذیه سالم مصرف 5 بار میوه و سبزیجات در روز می باشد. با توجه به اهمیت شناخت عوامل موثر بر مصرف میوه و سبزیجات در دختران نوجوان، پژوهش حاضر با هدف بررسی میزان تبعیت دختران دبیرستانی از برنامه پنج نوبت مصرف میوه و سبزیجات بر اساس تئوری رفتار برنامه ریزی شده، انجام گرفت. مواد و روش ها: در این مطالعه توصیفی- تحلیلی، 400 دانش آموز دختر دبیرستان های شهر همدان به روش نمونه گیری خوشه ای چند مرحله ای انتخاب شدند. در ابتدا شرکت کنندگان پرسشنامه مربوط به متغیرهای جمعیت شناختی و تئوری رفتار برنامه ریزی شده را تکمیل کردند، سپس در فاصله یک هفته بعد پرسشنامه BRFSS را تکمیل نمودند. داده ها با نرم افزار SPSS-18 و با استفاده از آزمون های کای دو، همبستگی پیرسون و رگرسیون لجستیک مورد تجزیه و تحلیل قرار گرفت.  یافته ها: میزان مصرف میوه و سبزیجات توسط دانش آموزان دختر 3.4 بار در روز بود و بعد خانوار، تحصیلات مادر، شغل پدر، درآمد خانوار، نمایه توده بدنی و نوع مدرسه با مصرف میوه و سبزیجات رابطه معنی داری داشت (0.05>P). قصد رفتاری 35 درصد از رفتار مصرف میوه و سبزیجات را پیش بینی می نمود، همچنین هنجارهای انتزاعی، کنترل رفتاری درک شده و نگرش 32 درصد از قصد رفتاری را پیش بینی می کردند.   نتیجه گیری: مصرف میوه و سبزیجات در دانش آموزان دختر در حد توصیه شده نبود. تئوری رفتار برنامه ریزی شده می تواند در طراحی مداخلات مصرف 5 بار میوه و سبزیجات جهت دانش آموزان دختر موثر باشد.

Title: رفتارهای خودمراقبتی ارتقاء دهنده سلامت و عوامل مرتبط با آن در سالمندان: کاربرد مدل اعتقاد بهداشتی

http://jech.umsha.ac.ir/article-1-47-fa.html

مقدمه: باورهای بهداشتی به طور قابل توجهی در اتخاذ رفتارهای خودمراقبتی ارتقادهنده سلامت تاثیر گذار هستند. یکی از شناخته شده ترین مدل های مبتنی بر باور، مدل اعتقاد بهداشتی است. پژوهش حاضر با هدف بررسی ارتباط بین سازه های این مدل و برخی عوامل دموگرافیک با رفتارهای خودمراقبتی ارتقاء دهنده سلامت در سالمندان انجام گردید. مواد و روش ها: این مطالعه توصیفی تحلیلی با مشارکت 465 نفر از سالمندان مراجعه کننده به فرهنگسراهای شهر تهران با استفاده از روش نمونه گیری چند مرحله ای انجام شد. ابزارهای مورد استفاده شامل پرسشنامه های دموگرافیک، سنجش باورهای بهداشتی، خودکارآمدی و رفتارهای خودمراقبتی ارتقاء دهنده سلامت بود. داده های جمع آوری شده با استفاده از نرم افزار SPSS ویرایش 22 و بر اساس آزمون های تی مستقل، آنالیز واریانس یکطرفه، همبستگی پیرسون و رگرسیون خطی چند متغیره مورد تجزیه و تحلیل قرار گرفت.  یافته ها: میانگین (±انحراف معیار) سنی آزمودنی ها 6.12±68.24 سال و میانگین نمره کلی رفتارهای خودمراقبتی 0.36±1.79 بود. جنسیت(0.011=P)، وضعیت اقتصادی(0.001>P)، سطح تحصیلات(0.001>P) و سن(0.0 08=P) به طور معناداری با رفتارهای خودمراقبتی ارتباط داشتند. بر پایه تحلیل رگرسیونی مدل اعتقاد بهداشتی موانع درک شده، خودکارآمدی و شدت درک شده به عنوان تعیین کننده های نهایی رفتار مشخص شدند(0.001>P).  نتیجه گیری: با توجه به نتایج این پژوهش، ضروری است که در طراحی برنامه های ارتقاء سلامت سالمندان بر افزایش خودکارآمدی و شدت درک شده و همچنین بر کاهش موانع درک شده توجه ویژه ای شود.

Title: تبیین احساس تنهایی در سالمندان و تحلیل آن بر اساس تئوری رشد روانی-اجتماعی: یک مطالعه کیفی

http://jech.umsha.ac.ir/article-1-41-fa.html

مقدمه: تنهایی بطور کلی یک حس ذهنی است که در همه انسانها وجود دارد. اما بسیاری از مردم ممکن است از صحبت در مورد آن، بدلیل برچسب خوردن احساس شرم داشته باشند. این مطالعه با هدف تبیین احساس تنهایی در سالمندان و تحلیل آن با تئوری رشد روانی-اجتماعی اریکسون انجام گرفته است. مواد و روش ها: این مطالعه به صورت کیفی و با رویکرد تحلیل محتوی انجام شد. جامعه پژوهش شامل 12 نفر از سالمندان 85-68 سال، مقیم شهر همدان بودند. نمونه گیری به صورت هدفمند شروع و با اشباع داده ها خاتمه یافت. برای جمع آوری داده ها از مصاحبه استفاده شد. تمامی مصاحبه ها ضبط و بلافاصله دست نویس شدند و داده ها با استفاده از تحلیل محتوی به روش قراردادی تجزیه و تحلیل گردید.  یافته ها: 187 کد اولیه از داده ها بدست آمد، پس از ادغام کدهای مشابه و دسته بندی آنها 7 زیر طبقه مفهوم سازی گردید و از زیر طبقات سه طبقه اصلی از داده ها استخراج شد. طبقات اصلی شامل: احساس بیگانه شدن، دوری از فعالیتهای مورد علاقه و احساس خستگی و غم بود. درونمایه مشترک طبقات حسرت توام با دلتنگی بود.  نتیجه گیری: با توجه به اینکه تنهایی در افراد مسن رایج است و با پیامدهای بهداشتی منفی از نظر سلامت جسمی و روانی همراه است، اتخاذ استراتژی های مداخله ای مناسب با استفاده از تئوری رشد روانی- اجتماعی اریکسون، موجب کاهش تنهایی و بهبود کیفیت زندگی سالمندان و حس ارزشمندی در این دوره زندگی می گردد.

Title: خودکارآمدی مربوط به انتخاب نوع زایمان و زمان لیبر در زنان باردار مراجعه کننده به مراکز بهداشتی درمانی شهرستان پارس آباد

http://jech.umsha.ac.ir/article-1-42-fa.html

مقدمه: خودکارآمدی باور شخص به توانایی خود جهت انجام موفقیت آمیز رفتار است. در رابطه با انتخاب نوع زایمان، خودکارآمدی به توانایی فرد جهت مقابله با شرایط استرس زا و انجام واکنش های ضروری در زمان زایمان مربوط می شود. مطالعه حاضر با هدف تعیین تأثیر خودکارآمدی در انتخاب نوع زایمان و زمان لیبر در زنان باردار انجام گردید. مواد و روش ها: مطالعه توصیفی- مقطعی حاضر در سال 1393 در بین 200 مادر باردار شکم اول و دوم با زایمان طبیعی قبلی تحت پوشش 5 مراکز بهداشتی-درمانی شهرستان پارس آباد انجام شد. زنان مورد مطالعه با روش نمونه گیری آسان انتخاب شدند. گردآوری اطلاعات با استفاده از پرسشنامه شامل مشخصات جمعیت شناختی، خودکارآمدی انتخاب زایمان طبیعی و خودکارآمدی زمان لیبر به روش خود گزارش دهی بود. داده های جمع آوری شده با نرم افزار SPSS-21 و با استفاده از آزمون های کای دو، آزمون دقیق فیشر و تی مستقل مورد تجزیه و تحلیل قرار گرفت.  یافته ها: میانگین سنی مادران 4.48±25.19 بود؛ بین تحصیلات همسر، داشتن بیمه تکمیلی و شغل با انتخاب روش زایمان ارتباط معنی داری وجود داشت (0.05>P). خودکارآمدی انتخاب زایمان طبیعی و زمان لیبر با تعداد زایمان و انتخاب روش زایمان ارتباط داشتند (0.001>P). میانگین خودکارآمدی در مادران باردار شکم دوم و افرادی که زایمان طبیعی را انتخاب کرده بودند، بیشتر بود.  نتیجه گیری: خودکارآمدی انتخاب روش زایمان و زمان لیبر در انتخاب روش زایمان تعیین کننده و با تعداد زایمان مرتبط می باشد؛ لذا پیشنهاد می گردد در مداخلات آموزشی بر خودکارآمدی جهت کاهش ترس از زایمان به ویژه در مادران باردار شکم اول توجه گردد.

Title: بررسی سواد سلامت بزرگسالان شهرستان تویسرکان

http://jech.umsha.ac.ir/article-1-37-fa.html

مقدمه: سواد سلامت، ظرفیت افراد برای کسب، پردازش و درک اطلاعات و خدمات بهداشتی پایه مورد نیاز برای تصمیم گیری های سلامتی می باشد. این مطالعه با هدف تعیین سطح سواد سلامت بزرگسالان شهرستان تویسرکان طراحی و اجرا گردید.  مواد و روش ها: این پژوهش، یک مطالعه توصیفی- تحلیلی از نوع مقطعی بود که در سال 1393 با مشارکت 285 نفر از افراد 60-20 ساله مراجعه کننده به مراکز بهداشتی شهرستان تویسرکان با استفاده از روش نمونه گیری در دسترس انجام شد. جهت سنجش سواد سلامت، از پرسشنامه سواد سلامت ایرانیان استفاده گردید. داده‌ها با استفاده از نرم‌افزار Stata-11 و با آزمون تی مستقل و آنالیز واریانس یکطرفه مورد تجزیه و تحلیل قرار گرفت. یافته ها: سن اکثریت افراد بین گروه سنی 20 تا 30 (52.3 درصد) بود. 53.7 درصد از شرکت کنندگان مرد و سطح تحصیلات اغلب آنها دیپلم (55.8 درصد) و شغل غالب آنها دانشجو (31.9 درصد) بود. شرکت کنندگان در دسترسی به اطلاعات ضعیف (42.1 درصد)، در درک اطلاعات ضعیف (54.4 درصد)، در قضاوت و ارزیابی متوسط (64.9 درصد) و در استفاده از اطلاعات در سطح متوسط (88.8 درصد) بودند.   نتیجه گیری: بطورکلی سواد سلامت در افراد نامطلوب بود. این موضوع توجه بیشتر به سواد سلامت را در برنامه های آموزش و ارتقاء سلامت نشان می دهد. لازم است برای بزرگسالانی که از سواد سلامت نامطلوب برخوردارند، طی برنامه های جامع، رسانه ها و مواد آموزشی ساده و قابل فهم طراحی و بکار رود.

Title: تأثیر برنامه آموزشی مبتنی بر مدل اعتقاد بهداشتی در کاهش استفاده از تلفن همراه هنگام رانندگی در بین رانندگان تاکسی

http://jech.umsha.ac.ir/article-1-33-fa.html

مقدمه: استفاده از تلفن همراه هنگام رانندگی به یک عامل تهدید کننده امنیت ترافیکی تبدیل شده است. مطالعه حاضر با هدف تعیین تأثیر برنامه آموزشی مبتنی بر مدل اعتقاد بهداشتی در کاهش استفاده از تلفن همراه هنگام رانندگی در بین رانندگان تاکسی شهر تویسرکان انجام شد.   مواد و روش‌ها: در این مطالعه مداخله‌ای نیمه تجربی، 110 نفر از رانندگان تاکسی زیر 35 سال شهر تویسرکان به‌صورت تصادفی و مساوی در دو گروه مداخله و کنترل قرار گرفتند. ابزار جمع‌آوری داده‌ها پرسشنامه‌ای شامل سازه‌های مدل اعتقاد بهداشتی، سؤالات آگاهی، رفتارهای استفاده از تلفن همراه و متغیرهای زمینه‌ای بود که به روش خودگزارش‌دهی تکمیل گردید. مداخله آموزشی در گروه مداخله شامل 3 جلسه آموزشی 90 دقیقه‌ای بود. دو ماه پس از انجام مداخله، هر دو گروه پیگیری شدند. تجزیه و تحلیل داده ها با استفاده از نرم افزار spss-19 و بکارگیری آزمون‌های کای‌دو، تی مستقل، تی زوجی و مک‌نمار صورت گرفت.   یافته‌ها: در میانگین نمرات سازه‌های مدل اعتقاد بهداشتی (حساسیت، شدت، موانع، منافع درک شده، خودکارآمدی و راهنما برای عمل)، آگاهی، رفتارهای مطلوب عدم استفاده از تلفن همراه هنگام رانندگی، قبل از مداخله آموزشی بین دو گروه تفاوت معنی‌داری وجود نداشت، اما بعد از مداخله آموزشی، در گروه مداخله اختلاف معنی‌داری نسبت به گروه کنترل مشاهده گردید. میزان استفاده از تلفن همراه در گروه مداخله بعد از مداخله آموزشی 35.14 درصد کاهش یافت.  نتیجه گیری: مداخله آموزشی براساس مدل اعتقاد بهداشتی می‌تواند سبب کاهش استفاده از تلفن همراه هنگام رانندگی در رانندگان تاکسی گردد.

Title: کاربرد الگوها و تئوری‌های آموزش و ارتقاء سلامت در مداخلات فعالیت جسمانی برای زنان: یک مرور نظام مند

http://jech.umsha.ac.ir/article-1-34-fa.html

مقدمه: پژوهش حاضر، مداخلات انجام شده مبتنی بر الگوها و تئوری‌های آموزش و ارتقاء سلامت را در زمینۀ فعالیت جسمانی زنان به شیوۀ نظام‌مند مورد بررسی و تحلیل قرار داده است. مواد و روش‌ها: 3 پایگاه اطلاعاتی الکترونیک شامل Springer، Biomed central و Science Direct به شیوۀ نظام‌مند مورد بررسی قرار گرفت. از بین پژوهش ها، مطالعاتی انتخاب شدند که کمی، مداخله‌ای و به زبان انگلیسی بوده و حداقل یکی از الگوها و تئوری‌های آموزش و ارتقاء سلامت را بکار برده بودند. در نهایت 13 مطالعه دارای معیار ورود به مطالعه از ابتدای سال 2000 تا پایان 2013 میلادی در مطالعه مورد بررسی قرار گرفتند. یافته‌ها: از 13 مطالعۀ بررسی شده، 10 مطالعه میزان فعالیت جسمانی را قبل و بعد از مداخله سنجیده بودند که 9 مداخله باعث افزایش معنی‌دار فعالیت جسمانی در گروه مداخله نسبت به گروه کنترل شده بود. مطالعات در محیط‌های ارتقاء سلامت شامل مراکز مراقبت سلامت، محیط اجتماعی محل کار انجام شده بود. پرکاربردترین الگوی مورد استفاده، الگوی فرانظری بود که در 8 مطالعه مبنای مداخله بود.   نتیجه گیری: پیشنهاد می‌شود به‌منظور تاثیرگذاری بیشتر مداخلات، تمرکز مداخله روی فعالیت جسمانی اعمال شده و طول دوره مداخله افزایش یابد. در مداخلات مبتنی بر الگوی فرانظری پیشنهاد می‌شود مراحل تغییر رفتار فعالیت جسمانی در گروه‌های مداخله و کنترل اندازه‌گیری گردد تا یک مقیاس تکمیلی برای سنجش میزان تاثیر مداخله فراهم شود. طبق نتایج، هیچکدام از مطالعات، ایجاد تغییر در سیاست‌های سازمانی یا عمومی سلامت و یا ایجاد تغییرات در محیط فیزیکی مرتبط با فعالیت جسمانی را هدف قرار نداده بودند.
